# Pulmonary Arterial Hypertension Due to Talc Granulomatosis Following Intravenous Use of Oral Medications: A Report of a Rare Case

**DOI:** 10.7759/cureus.88887

**Published:** 2025-07-28

**Authors:** Keerthana Nagarajan, Jacinth Preethi, Sathya A, Rangaswami M, Chandrasekar Selvaraj

**Affiliations:** 1 Department of General Medicine, Government Stanley Medical College & Hospital, Chennai, IND

**Keywords:** centrilobular nodules, intravenous drug abuse, pulmonary arterial hypertension, pulmonary talcosis, tapentadol

## Abstract

Pulmonary talcosis is a rare granulomatous lung disease resulting from the intravenous injection of crushed oral tablets containing insoluble excipients such as talc, microcrystalline cellulose, and crospovidone. These substances embolize within the pulmonary vasculature, provoking granulomatous inflammation that may progress to pulmonary arterial hypertension (PAH). We present a case report of a 25-year-old male who presented with acute-onset shortness of breath, chest pain, dry cough, fatigue, and palpitations. On examination, he was dyspneic and tachypneic and exhibited bilateral basal inspiratory crackles with signs of right heart strain. Imaging revealed elevated right ventricular pressures and computed tomography (CT) findings of centrilobular nodules with sparing of the subpleural regions and fissures, characteristic of talc granulomatosis. A detailed history revealed longstanding intravenous polysubstance abuse over five years, including methamphetamine, tapentadol, and cocaine, likely contributing to his pulmonary pathology. Based on clinical, imaging, and echocardiographic findings, a diagnosis of pulmonary hypertension secondary to talcosis was established. The patient was managed with supplemental oxygen, antiplatelet therapy, and oral sildenafil, resulting in symptomatic improvement, and was strongly advised to cease intravenous drug use. A lung biopsy was planned for histological confirmation. This case report aims to emphasize the importance of recognizing pulmonary talc granulomatosis as a rare but serious complication of intravenous drug use, particularly in patients presenting with unexplained respiratory symptoms, due to its potential to cause irreversible PAH if left untreated.

## Introduction

Pulmonary talcosis is a rare form of foreign body granulomatosis caused by the intravenous injection of crushed tablets intended for oral administration and has been observed in approximately 5% of intravenous drug users [1]. Several medications containing talc, including methylphenidate (Ritalin), methadone, tripelennamine (Pyribenzamine), propoxyphene (Darvon), phenmetrazine (Preludin), and amphetamines, have been implicated in its pathogenesis. These tablets typically contain insoluble binding agents such as talc (hydrated magnesium silicate), microcrystalline cellulose, and crospovidone. These substances become irreversibly trapped in the lungs and induce angiocentric foreign body granulomatous inflammation [2,3]. The clinical manifestations depend on the severity of arteriolar involvement and the extent of resulting pulmonary arterial hypertension (PAH). Patients may initially be asymptomatic and later develop nonspecific symptoms, such as dry cough, chest pain, and exertional breathlessness, as the disease progresses. Diagnosing pulmonary talc granulomatosis is challenging due to overlapping clinical and radiographic findings [4,5]. A thorough patient history, particularly regarding substance use, and a high index of suspicion are essential for diagnosis.

## Case presentation

A 25-year-old male with no known comorbidities or family history presented to our hospital with complaints of shortness of breath, chest pain, dry cough, fatigue, and palpitations for the past three days. He had a five-year history of intravenous polysubstance abuse, including methamphetamine, tapentadol, and cocaine.

On clinical examination, the patient was conscious, oriented, afebrile, dyspneic at rest, and tachypneic. There was no pallor, icterus, cyanosis, clubbing, or pedal oedema. Multiple tattoo marks were observed all over the body, along with track marks over the left antecubital fossa. The heart rate was 108/min, respiratory rate 22/min, blood pressure 100/60 mmHg, SpO2 94% on room air (98% on 4 L oxygen), and capillary blood glucose 126 mg/dL. Respiratory system examination revealed bilateral basal inspiratory crackles. Cardiovascular examination revealed elevated jugular venous pressure, a right parasternal heave, a palpable P2, and a loud P2 on auscultation. Other systemic examinations were unremarkable.

Laboratory investigations showed a normal complete blood count, renal and liver function tests, and D-dimer levels, with negative cardiac biomarkers. Blood cultures were sterile, and viral screening was positive for hepatitis B but negative for hepatitis C and HIV. Autoimmune panels showed no evidence of connective tissue disease. Arterial blood gas analysis was within normal limits. Pulmonary function tests indicated a mild restrictive pattern with decreased diffusion capacity.

**Figure 1:**
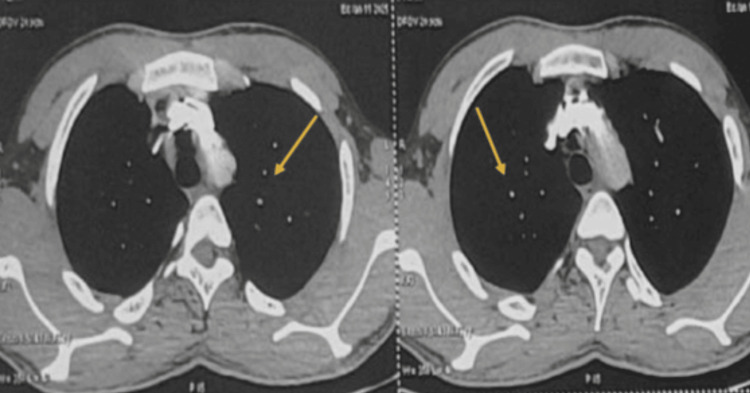
Axial contrast-enhanced CT of the thorax (mediastinal window) from CT pulmonary angiogram (CTPA) showing centrilobular nodules with subpleural and fissural sparing in both lungs (arrows), suggestive of talc granulomatosis. CT: computed tomography

Chest X-ray revealed cardiomegaly. Computed tomography (CT) chest screening showed diffuse centrilobular nodules with subpleural and fissural sparing in both lungs, along with ground-glass opacities in the anterior segment of the right upper lobe, the apicoposterior segment of the left upper lobe, and the superior segment of the left lower lobe- findings suggestive of talc granulomatosis. Figure 1 demonstrates corresponding axial mediastinal window images from the contrast-enhanced CT pulmonary angiogram (CTPA), which confirmed the presence of hyperdense centrilobular nodules.

**Figure 2:**
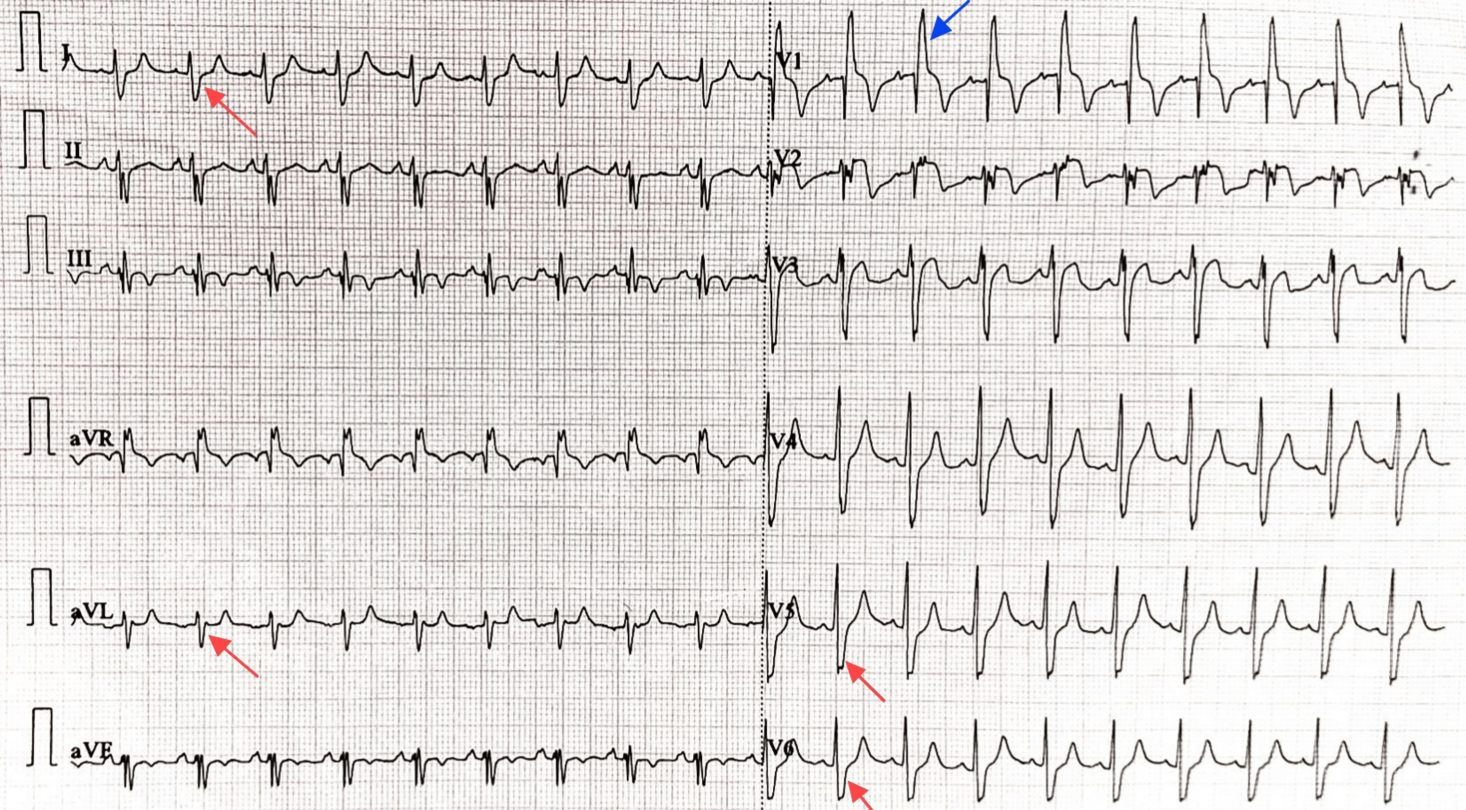
Twelve-lead ECG demonstrating sinus tachycardia with rSR' pattern in V1 and wide, slurred S waves in lateral leads (I, aVL, V5, V6), suggestive of RBBB. ECG: electrocardiogram; RBBB: right bundle branch block

Electrocardiogram revealed sinus tachycardia, right bundle branch block, and right ventricular strain patterns (Figure 2).

Echocardiography demonstrated right atrial and right ventricular enlargement with systolic dysfunction, moderate tricuspid regurgitation, a tricuspid regurgitant pressure gradient of 45 mmHg, and dilation of the main pulmonary artery (2.75 cm). The left ventricle appeared D-shaped, with an ejection fraction of 55%. No thrombus, vegetation, or pericardial effusion was noted. A bilateral lower limb venous Doppler study showed no evidence of deep venous thrombosis. CT pulmonary angiography was performed to evaluate for pulmonary embolism. The CT angiography study of the pulmonary arteries showed no evidence of thrombus in the main, right, or left pulmonary arteries or their branches.

Based on clinical, radiologic, and echocardiographic findings, a diagnosis of pulmonary hypertension secondary to pulmonary talc granulomatosis was made. Management included nasal oxygen therapy, antiplatelet agents, and oral sildenafil 20 mg three times daily. The patient showed symptomatic improvement and was advised to cease drug use. Lung biopsy was deferred in view of the patient’s stable condition and was planned as a potential future step pending disease progression or diagnostic uncertainty.

## Discussion

Pulmonary foreign body granulomatosis is a rare pulmonary disorder resulting from the intravenous injection of crushed tablets intended for oral administration and has been observed in approximately 5% of intravenous drug users [1]. These tablets often contain insoluble binding agents such as talc (hydrated magnesium silicate), microcrystalline cellulose, crospovidone, potato starch, and cornstarch. Talc, commonly used as a filler and lubricant in medications like methadone, methylphenidate, amphetamines, and pentazocine, can lead to significant pulmonary complications when injected.

The intravenous injection of talc-containing drugs results in the embolization of talc particles into the pulmonary vasculature. This leads to an initial arteritis, followed by the development of foreign body granulomas as talc particles migrate through the vessel walls into the surrounding perivascular and pulmonary interstitial tissues. Consequently, this process can result in PAH and interstitial fibrosis [6]. The severity of clinical symptoms correlates with the extent of arteriolar involvement and the degree of PAH. Patients may present with nonspecific symptoms such as dry cough and exertional dyspnoea. In advanced cases, manifestations may include cor pulmonale, panlobular emphysema [7], spontaneous pneumothorax, and acute respiratory distress syndrome (ARDS).

Pulmonary function tests often reveal a reduction in the diffusion capacity for carbon monoxide. Chest radiography may be normal; however, high-resolution computed tomography (HRCT) can demonstrate nodular opacities, diffuse ground-glass patterns, and lower lobe-predominant panacinar emphysema [8,9]. Echocardiography is useful for assessing pulmonary artery pressures and ventricular function. In our case, echocardiographic findings were consistent with significant pulmonary hypertension. While right heart catheterization remains the gold standard [10-12], it was deferred in this case due to the patient’s stable clinical condition and the high degree of diagnostic certainity based on non-invasive findings. CT pulmonary angiography is also performed to rule out thromboembolic disease. Differential diagnoses such as pneumoconiosis, sarcoidosis, interstitial lung disease, lymphangitic carcinomatosis, miliary tuberculosis, opportunistic infections, acute pulmonary thromboembolism, and septic embolism should be considered and appropriately excluded. When the diagnosis remains unclear, bronchoscopy and lung biopsy may be warranted. Histopathology typically reveals perivascular and intravascular clusters of foreign material within granulomas. Talc crystals are described as needle-shaped or plate-like and are birefringent under polarized light [13].

A large forensic study by Darke et al. reviewed 373 autopsies of intravenous drug users in Sydney between 1997 and 2013 and identified a substantial increase in foreign body pulmonary embolization due to injection of crushed oral medications. Moderate to abundant embolic burden was seen in 43% of cases. Pulmonary hypertension and right-sided heart pathology were found in 10% and 5% of cases, respectively. The extent of embolization was significantly associated with these complications, underscoring the cardiopulmonary sequelae of intravenous tablet abuse [14].

Currently, there is no established specific treatment for foreign body granulomatosis. Management primarily involves supportive care and cessation of intravenous drug use. Asymptomatic patients should undergo periodic assessments to monitor disease progression. Although data supporting glucocorticoid therapy are limited, some reports suggest a potential benefit. For instance, a case report [15] described a patient who experienced rapid symptomatic improvement with a daily dose of 60 mg prednisolone. Pulmonary hypertension associated with this condition may be managed with vasodilators; in some cases, oral sildenafil has shown promise. However, treatment should be tailored to the individual, considering potential drug interactions and contraindications. Lung transplantation may be considered in patients with end-stage disease unresponsive to medical therapy [16]. It is important to note that most patients have a poor prognosis due to the progressive decline in pulmonary function.

Although several stimulants, such as anorexigens, methamphetamine, and cocaine, have been implicated as potential risk factors for the development of PAH [17], the radiological pattern in this case was more suggestive of talc granulomatosis. Given the characteristic imaging features and a history of intravenous use of crushed oral medications, talc granulomatosis was considered the most likely cause of pulmonary hypertension in this case, although stimulant use may have also contributed. This case report provides a comprehensive clinical evaluation and utilizes a range of diagnostic tests, including imaging studies and echocardiography, to diagnose pulmonary hypertension secondary to pulmonary talc granulomatosis in a young patient with a history of intravenous polysubstance abuse. The inclusion of various diagnostic modalities strengthens the reliability of the diagnosis. However, a limitation is the absence of histopathological confirmation via lung biopsy and the lack of right heart catheterisation, which remains the gold standard for diagnosing PAH. Talc exposure and stimulant use may have contributed synergistically to the development of pulmonary hypertension in this patient. Nevertheless, the patient’s symptomatic improvement with pulmonary vasodilator therapy further supports the diagnosis.

This study highlights the importance of integrating clinical presentation, laboratory findings, and characteristic CT thorax and echocardiographic features in diagnosing pulmonary talc granulomatosis by systematically excluding other potential causes of pulmonary hypertension.

## Conclusions

Patients with a history of intravenous drug use who present with unexplained dyspnea should be thoroughly evaluated for possible PAH and pulmonary talc granulomatosis. A comprehensive clinical assessment, including detailed history and appropriate imaging such as chest CT, is essential. In cases where diagnosis remains uncertain, bronchoscopy with biopsy and right heart catheterization should be considered to confirm histopathological and hemodynamic findings. In the absence of these, a presumptive diagnosis may be made based on clinical and radiological correlation. Currently, no specific treatment is established; management consists of supportive care and cessation of intravenous drug use. Lung transplantation may be considered in end-stage, refractory cases.
